# Association between Arsenic Exposure and Diabetes Mellitus in Cambodia

**DOI:** 10.1155/2014/683124

**Published:** 2014-05-18

**Authors:** Jhih-Wei Huang, Ya-Yun Cheng, Tzu-Ching Sung, How-Ran Guo, Suthipong Sthiannopkao

**Affiliations:** ^1^Department of Environmental and Occupational Health, College of Medicine, National Cheng Kung University, Tainan 70428, Taiwan; ^2^Department of Health Care Management, University of Kang Ning, Tainan 70970, Taiwan; ^3^Department of Occupational and Environmental Medicine, National Cheng Kung University Hospital, Tainan 70428, Taiwan; ^4^Department of Environmental Engineering, Dong-A University, Busan 604-714, Republic of Korea

## Abstract

Whereas studies in Taiwan found associations between arsenic exposure from drinking water and diabetes mellitus (DM), studies in other countries yielded inconsistent results, and diet might be a confounder. We conducted a study in Cambodia, where people have non-Western style diet, to evaluate the association. We measured well water and urine samples and examined skin signs of arsenicosis to assess arsenic exposure and used questionnaires to collect data on potential risk factors. We performed a fingertip blood glucose test followed by measurement of hemoglobin A1c to assess DM. The 43-male and 99-female participants had an average age of 40.4 years. We found that participants with skin signs of arsenicosis had a higher level of arsenic in the drinking water (1101.1 versus 972.2 **μ**g/L, *P* = 0.02). Drinking water with arsenic levels above the median (907.25 **μ**g/L) was associated with a nearly twofold increase in the risk of DM (odds ratio [OR] = 1.7, 95% confidence interval [CI]: 0.5–5.8), so was having skin sings of arsenicosis (OR = 1.7, 95% CI: 0.5–5.6). The ORs did not reach statistical significance most likely because of the small case number. Therefore, further studies with larger study populations are needed to confirm our findings.

## 1. Introduction

Arsenic is widely distributed in the nature and mainly transported in the environment by water. It can be found in both inorganic and organic forms in the natural environment, and inorganic forms of arsenic, which are the predominant forms in groundwater reservoirs, are more toxic than organic forms. Arsenic can easily be released from soil in to ground water, depending on the temperature, pH, oxidation reduction potential, dissolved oxygen, and conductivity.

In the past several decades, the association between arsenic and cancers has been well-documented [1–9]. In addition, other arsenic-related diseases were identified, including diabetes mellitus (DM) [10–13], which is one of the most common chronic diseases in the world. Non-insulin-dependent (type 2) DM is the dominating form, which is a metabolic disorder characterized by hyperglycemia, insulin resistance in peripheral tissues, and altered insulin secretary capacity of pancreatic *β* cells. Type 2 DM accounts for 90–95% of all cases and is a major public health problem worldwide. In general, DM cannot be cured and may lead to complex complications, even death, without treatment.

In the past several decades, epidemiologic studies have found that arsenic exposure may increase the risk of DM, including those in Taiwan [12], Mexico [14], and Bangladesh [13]. However, such an association was not observed in some studies in developed countries such as USA [15], Spain [16], and England [17]. Because diet is a major risk facor of DM, differneces in the diet might account for the inconsistency in study results and acted as a confounder. Some areas in East Asia countries such as Vietnam, Liaos, and Cambodia were found to have high levels of arsenic in the ground water, and most of these countries are developing countries where groundwater is the main source of drinking water or daily water usage. Because people in these countries generally have non-Western style diet, studies in these countries can minimize confounding effects caused by traditional risk factors, including diet. Therefore, we conducted a study in Cambodia to assess the association between arsenic exposure and DM. For many people living in Cambodia, especially in rural areas, groundwater is the only source of drinking water, and in some areas where surface water is also used for drinking water, groundwater from tube wells, which is considered as relatively free of pathogens, is the main sources of drinking water. However, high arsenic levels were found in the drinking water in some of these areas.

## 2. Materials and Methods

### 2.1. Study Area and Population

Cambodia, officially known as the Kingdom of Cambodia, is located in the southern portion of the Indochina Peninsula in Southeast Asia and has a total landmass of 181,035 km^2^. High levels of arsenic in the ground water were found in some areas such as Kandal and Prey Veng, and the highest arsenic level, up to 907 *μ*g/L, was found in a remote village in Kandal called Preak Russey [18]. In the Kandal Province, about 1 million people had stopped using surface water or water from shallow wells due to the prevalence of bacterial diseases, and it has been a common practice for years for residents to pump groundwater from private tube wells.

We recruited participants in the Preak Russey Village at a survey station. Information of the recruitment had been distributed to the residents beforehand. Residents of the village generally have regular life styles and consistent diet, and rice and corn are the staple foods.

### 2.2. Questionnaire Survey

We collected information from each participant through a personal questionnaire, which included questions on demographic characteristics such as age, gender, income, education level, and occupation. We also collected data on body size, smoking habit, family history of chronic disease, and persona medical history, particularly the diagnosis of DM. In addition, to help the assessment of arsenic exposure, we obtained the address of the participant and the duration of residence in the study area and asked about the source of drinking water and signs of arsenicosis.

### 2.3. Assessment of Arsenic Exposure

We assessed participants' arsenic exposure using three indicators: arsenic concentration in the drinking water, arsenic level of the urine, and skin signs of arsenicosis. We obtained a freshly void sample of urine from the participant right after the interview. Urine samples were first collected in a plastic cup and then put into two 50 mL bottles for shipment. For each tube well used by participants, we located the well and recorded its location using GPS during sampling. Before sampling, we conducted 5 to 10 minutes of flushing using the well water to remove any sandy water. After the sampling, all of the collected samples were kept in an ice box and then transferred to a refrigerator where they were stored at 4°C until before delivery.

Measurements of the arsenic concentrations in the water samples were performed at the Gwangju Institute of Science and Technology (GIST) in South Korea using inductively coupled plasma mass spectrometry (ICP-MS, Agilent 7500ce). ICPMS with traditional sample introduction (direct nebulization) can determine total arsenic levels as low as 0.2 *μ*g/L. The sample was acidified and sprayed (via a nebulizer) into an argon plasma, and the high temperature of the plasma atomized and ionized all forms of arsenic. By doing so, the response does not vary across species as with more traditional atomic absorption (AA) and graphite furnace atomic absorption (GFAA) methods, which require thorough digestion prior to analysis. Urine arsenic levels were determined using anion-exchange HPLC/ICPMS, also at the GIST.

### 2.4. Definition of Hyperglycemia

According to the American Diabetes Association (ADA) [19], DM can be diagnosed by one of following criteria.

1. Fasting blood glucose ≥ 126 mg/dL.
2. Random blood glucose ≥ 200 mg/dL together with classic symptoms of hyperglycemia or hyperglycemic crisis.
3. Two-hour glucose ≥ 200 mg/dL in an oral glucose tolerance test (OGTT) with 75 g glucose.
4. Hemoglobin A1c (HbA1c) ≥ 6.5%.

In our study, we adopted the first and fourth criteria and used fingertip blood glucose instrument to measure participants' fasting glucose levels for screening hyperglycemia with a cut-off of 100 mg/dL. When a participant's blood glucose level was greater than 100 mg/dL, we took a blood sample and measured the level of HbA1c to confirm the diagnosis of DM. All blood samples were taken by a local nurse and stored with ice in the ice box. We delivered the samples to a local laboratory for analyses when we finished our field work each day. Participants who were under medical treatment for DM were also defined as cases of DM.

### 2.5. Statistical Analysis

We used Student's *t*-test and chi-square test to evaluate the differences in categorical and continuous variables, respectively, between two groups. Logistic regression was used for assessing the associations between arsenic exposure and DM. All statistical tests were performed at a two-tailed significant level of 0.05. Microsoft Excel and SPSS 17.0 were used to manage and analyze the data.

## 3. Results

A total of 142 local residents participated in the study, including 43 men and 99 women. The average age was 40.4 years, with a standard deviation (SD) of 18.8 years. Study participants were relatively lean and had a mean body mass index (BMI) of 19.7 kg/m^2^, with a SD of 3.2 kg/m^2^. The participants had lived in the village for an average of 36.8 (SD = 7.4) years and used ground water for an average of 14.1 (SD = 5.9) years.

Of the participants, 14 were found to have DM. In comparison with the other 128 participants who did not have DM (the reference group), DM cases were older (57.6 versus 38.6 years old, *P* < 0.01) and had lived in the village for a longer period (50.9 versus 34.5 years, *P* < 0.01) (Table 1). The distribution of smoking status was different (*P* = 0.03), with the DM cases having higher prevalence of current smokers (57.1% versus 26.6%).

The urine arsenic levels of the participants ranged from 4.95 to 324.90 *μ*g/L, with a mean of 68.88 (SD = 54.33) *μ*g/L and a median of 52.05 *μ*g/L. Cases of DM had a higher arsenic level in the urine (73.3 versus 68.4 *μ*g/L), but the difference did not reach statistical significance (*P* = 0.75). Using the arsenic level in urine as the exposure indicator, we separated participants into high and low exposure groups by adopting the median arsenic level as the cut-off. None of the differences between the two groups reached statistical significance (Table 2). The prevalence of DM was similar between the two groups, with 8.5% in the high exposure group versus 11.3% in the low exposure group, and the odds ratio (OR) of DM associated with the high exposure group was 0.7, with a 95% confidence interval (CI) of 0.2 to 2.2 (*P* = 0.58).

Using the arsenic level in drinking water as the exposure indicator, we did not find a significant difference between DM cases and the reference group (981.1 versus 1058.9 *μ*g/L, *P* = 0.22). When the participants were separated into high and low exposure groups with the median arsenic level (907.25 *μ*g/L) as the cut-off, we found there were more participants in the high exposure group with daily income less than 3 USD (93.0% versus 82.1%, *P* = 0.05) (Table 3). None of the other differences between the two groups reached statistical significance. The prevalence of DM was higher in the high exposure (11.6% versus 7.1%), with an OR of 1.7 (95% CI: 0.5–5.8), but the difference did not reach statistical significance (*P* = 0.39).

Using the skin signs of arsenicosis as the exposure indicator, we found that participants with the signs were older (46.2 versus 31.2 years old, *P* < 0.01) and lived in the village longer (41.2 versus 28.1 years, *P* < 0.01) (Table 4). They were also taller (155.3 versus 147.7 cm, *P* < 0.01) and heavier (49.6 versus 41.4 kg, *P* < 0.01) and had a larger BMI (20.5 versus 18.5 kg/m^2^, *P* < 0.01). The proportion of women was higher (75.9% versus 60.0%), with a marginal statistical significance (*P* = 0.06). Participants with the signs had a higher level of arsenic in the drinking water (1101.1 versus 972.2 *μ*g/L, *P* = 0.02) on average. They also had higher prevalence of DM (11.5% versus 7.3%), with an OR of 1.7 (95% CI: 0.5–5.6), but the difference did not reach statistical significance (*P* = 0.42).

## 4. Discussions

In our study, we used arsenic levels in urine, arsenic levels in drinking water, and skin signs of arsenicosis as exposure indicators. The concentration of total arsenic levels in urine has often been used as an indicator of recent arsenic exposure whether by inhalation or by ingestion because urine is the main route of excretion of most arsenic species. According to a review article [20], there was a significant correlation between arsenic levels in urine and arsenic levels in drinking water, which means that urine arsenic could be a good indicator for arsenic exposure. However, our study did not find a higher prevalence of DM in participants with urine arsenic levels higher than the median, which is different from some previous epidemiology studies [14, 21–25]. There are several possible reasons. First of all, the raining season for Cambodia is from May to October, and our sampling was between July and August, which was during the raining season. In raining season, most of the residents take surface water instead of ground water, and the half life of arsenic in urine is approximately 24 hours, which means it can reflect only the exposure one or two days earlier. In addition, our study measured the total arsenic in urine only, and many previous epidemiology studies had measured other species of arsenic such as arsenic III, arsenic V, dimethylarsinic acid (DMA), and monomethylarsonic acid (MMA). The level of total arsenic is more likley to be affected by the consumption of seafood, which increase the level of organic arsenic that is less toxic. Furthermore, we did not measure the level of creatinine and use the arsenic to creatinine ratio, which adjusts for the clearance of arsenic and thus is generally regarded as a better indicator than the arsenic level itself.

Measurement of total arsenic in drinking water is often used to assess arsenic exposure, and because most arsenic in ground water is in inorganic forms, the level is a better indicator for exposure to inorganic arsenic. In our study population, with an average age of around 40 years old and an average residential history of around 36 years at the same location, we can infer that people living in the Preak Russey Village have stable exposure to arsenic from well water near their houses. According to our study results, there was a positive association between arsenic levels in well water and DM, although the OR did not reach statistical significance. Judging from the large OR (1.7), we believe this was most likely due to the small case number. In addition, this finding is consistent with the findings in many previous epidemiology studies [12, 25–32].

Typical skin signs of arsenicosis should be a good indicator for chronic exposure to arsenic, because it reflects the biological effects in addition to exposure. Arsenicosis has been studied as an adverse outcome or used as a case definition in some epidemiology studies [33–35]. However, studies using this indicator to assess the association between arsenic exposure and DM are rare. In our study, in addition to a positive association between arsenicosis skin signs and DM, we found an association between skin signs of arsenicosis and arsenic levels in drinking water. This provides the evidence that skin signs of arsenicosis can be a good indicator of long term arsenic exposure.

In our study, there were more female participants than male participants. The main reason was that men have to work in field, especially for the young generation. During our sampling, the participation was voluntary, and the main reason for refusing participation was that the candidate needed to go farming within the time of our sampling.

We applied a two-step approach to evaluation of the existence of DM. In the first step, a screening was conducted using the level of fingertip blood glucose, and we adopted a cut-off of 100 mg/dL for fasting glucose. As the ADA defined DM using 126 mg/dL as the cut-off for fasting glucose, we used HbA1c level in the second step and adopted the ASA cut-off of 6.5%. Fasting glucose is affected by many factors, and DM cases may have levels lower than 126 mg/dL if they are under well diet control. HbA1c is an indicator of the long term glucose level in the past three months and thus is more stable than blood glucose screening. Using this two-step approach, we have defined DM in concordance with the ADA criteria and minimized the chance of underdiagnosis.

The small case number was the main limitation of our study. During the survey, participants joined the study at their own will, and in addition to the fact that many candidates had to work on the farm, the distance between the residence and survey station was a problem which may lower the willingness of participation. Although this problem might be overcome by house-to-house interview, roads in the Preak Russey Village were unpaved, which made this approach difficult to apply. Our survey was in the raining season, in which it usually rains in the afternoon, and we had to leave before it rained because cars could not run on the unpaved roads after raining. The transport of blood samples was also a potential limitation. In order to analyze HbA1c properly, we needed to deliver samples from the Preak Russey Village to a laboratory in Phnom Penh as soon as possible. Furthermore, we needed fasting fingertip blood glucose for screening, which means people could not come after they had finished their breakfast. During our sampling, we arrive at 7:00 am and leave around 10:30 am. The average work time was about two and half hours per day. Under above conditions, it was difficult to get a large sample size.

## 5. Conclusions

We observed an association between arsenic exposure from drinking water and DM, although the association did not reach statistical significance, most likely due to the small case number. We also demonstrate that skin signs of arsenicosis can be a good indicator of long term arsenic exposure. Further studies with larger population sizes are needed to confirm our findings.

## Figures and Tables

**Table 1:** Demographical data between the diabetes mellitus group and non-diabetes mellitus group.

	Diabetes mellitus	Non-diabetes mellitus	*P* value
	*N* = 14	*N* = 128	
Mean ± Standard Deviation			
Age (years)	57.6 (15.7)	38.6 (18.2)	<0.01
Height (cm)	151.1 (4.2)	152.5 (11.2)	0.33
Weight (kg)	45.9 (7.7)	46.5 (11.4)	0.83
Residence (years)	50.9 (18.2)	34.5 (16.5)	<0.01
Body mass index (kg/m^2^)	20.1 (3.3)	19.7 (3.2)	0.65
Urine arsenic level (*μ*g/L)	73.3 (59.1)	68.4 (54.0)	0.75
Water arsenic level (*μ*g/L)	981.1 (198.2)	1058.9 (358.1)	0.22
Numbers (%)			
Gender			0.55*
Male	3 (21.4)	40 (31.2)	
Female	11 (78.6)	88 (68.8)	
Education (years)			0.59*
None	8 (57.1)	52 (40.6)	
≤6	6 (42.9)	66 (51.6)	
7–9	0 (0.0)	8 (6.2)	
>9	0 (0.0)	2 (1.6)	
Smoking			0.03*
None	5 (35.7)	89 (69.5)	
Current	8 (57.1)	34 (26.6)	
Previous	1 (7.1)	5 (3.9)	
Income (USD per day)			0.51*
≤2	13 (92.9)	113 (88.3)	
3–9	1 (7.1)	15 (11.7)	
Arsenicosis			0.42
Negative	4 (28.6)	51 (39.8)	
Positive	10 (71.4)	77 (60.2)	

*Fisher's exact test.

**Table 2:** Characteristics between two groups of participants separated by the arsenic level in urine.

Arsenic level in urine	<52.03 *μ*g/L	≥52.03 *μ*g/L	*P* value
	*N* = 71	*N* = 71	
Mean ± Standard Deviation			
Age (years)	39.8 (20.0)	41.0 (17.7)	0.77
Height (cm)	152.2 (10.8)	152.6 (10.6)	0.81
Weight (kg)	45.2 (11.2)	47.7 (10.7)	0.17
Residence (years)	36.0 (18.8)	36.0 (16.0)	0.94
Body mass index (kg/m^2^)	19.1 (3.2)	20.8 (3.1)	0. 06
Water arsenic level (*μ*g/L)	1076.0 (357.1)	1026.3 (335.1)	0.39
Numbers (%)			
Gender			0.36
Male	19 (26.8)	24 (33.8)	
Female	52 (73.2)	47 (66.2)	
Education (years)			0.84
None	32 (45.1)	28 (39.4)	
≤6	35 (49.3)	37 (52.1)	
7–9	3 (4.2)	5 (7.1)	
>9	1 (1.4)	1 (1.4)	
Smoking			0.70
None	46 (64.8)	48 (67.6)	
Current	21 (29.6)	21 (29.6)	
Previous	4 (5.6)	2 (2.8)	
Income (USD per day)			>0.95
≤2	63 (88.7)	63 (88.7)	
3–9	8 (11.3)	8 (11.3)	
Arsenicosis			0.61
Negative	29 (40.8)	26 (36.6)	
Positive	42 (59.2)	45 (63.4)	
Diabetes mellitus			0.58
No	63 (88.7)	65 (91.5)	
Yes	8 (11.3)	6 (8.5)	

**Table 3:** Characteristics between two groups of participants separated by the arsenic level in drinking water.

Arsenic level in drinking water	<907.25 *μ*g/L	≥907.25 *μ*g/L	*P* value
	*N* = 56	*N* = 86	
Mean ± Standard Deviation			
Age (years)	40.7 (18.2)	40.3 (19.2)	0.89
Height (cm)	153.4 (7.6)	151.8 (12.2)	0.39
Weight (kg)	47.6 (9.4)	45.7 (11.9)	0.32
Residence (years)	37.4 (17.1)	35.2 (17.5)	0.47
Body mass index (kg/m^2^)	20.1 (3.2)	19.5 (3.2)	0.27
Urine arsenic level (*μ*g/L)	61.2 (44.9)	73.9 (59.4)	0.15
Numbers (%)			
Gender			0.69
Male	18 (32.1)	25 (29.1)	
Female	38 (67.9)	61 (70.9)	
Education (years)			0.45
None	25 (44.6)	35 (40.7)	
≤6	29 (51.8)	43 (50.0)	
7–9	1 (12.5)	7 (8.1)	
>9	1 (1.8)	1 (1.2)	
Smoking			0.16
None	34 (60.7)	60 (69.8)	
Current	21 (37.5)	21 (24.4)	
Previous	1 (1.8)	5 (5.8)	
Income (USD per day)			0.05
≤2	46 (82.1)	80 (93.0)	
3–9	10 (17.9)	6 (7.0)	
Arsenicosis			0.06
Negative	27 (48.2)	28 (32.6)	
Positive	29 (51.8)	58 (67.4)	
Diabetes mellitus			0.39
No	52 (92.9)	76 (88.4)	
Yes	4 (7.1)	10 (11.6)	

**Table 4:** Characteristics between two groups of participants separated by skin signs of arsenicosis.

Skin signs of arsenicosis	Positive	Negative	*P* value
	*N* = 87	*N* = 55	
Mean ± Standard Deviation			
Age (years)	46.2 (14.2)	31.2 (21.4)	<0.01
Height (cm)	155.3 (7.4)	147.7 (13.1)	<0.01
Weight (kg)	49.6 (8.5)	41.4 (12.5)	<0.01
Residence (years)	41.2 (14.0)	28.1 (19.1)	<0.01
Body mass index (kg/m^2^)	20.5 (2.8)	18.5 (3.4)	<0.01
Urine arsenic level (*μ*g/L)	72.4 (59.4)	63.4 (45.1)	0.34
Water arsenic level (*μ*g/L)	1101.1 (367.9)	972.2 (294.3)	0.02
Numbers (%)			
Gender			0.06
Male	21 (24.1)	22 (40.0)	
Female	66 (75.9)	33 (60.0)	
Education (years)			0.12
None	41 (47.1)	19 (34.5)	
≤6	38 (43.7)	34 (61.8)	
7–9	7 (8.0)	1 (12.5)	
>9	1 (1.1)	1 (1.8)	
Smoking			0.24
None	53 (60.9)	41 (74.5)	
Current	30 (34.5)	12 (21.8)	
Previous	4 (4.6)	2 (3.6)	
Income (USD per day)			0.91
≤2	77 (88.5)	49 (89.1)	
3–9	10 (11.5)	6 (10.9)	

## References

[1] Chen C-J, Chen CW, Wu M-M, Kuo T-L (1992). Cancer potential in liver, lung, bladder and kidney due to ingested inorganic arsenic in drinking water. *British Journal of Cancer*.

[2] Guo H-R (2003). The lack of a specific association between arsenic in drinking water and hepatocellular carcinoma. *Journal of Hepatology*.

[3] Guo H-R (2004). Arsenic level in drinking water and mortality of lung cancer (Taiwan). *Cancer Causes and Control*.

[4] Guo H-R, Tseng Y-C (2000). Arsenic in drinking water and bladder cancer: comparison between studies based on cancer registry and death certificates. *Environmental Geochemistry and Health*.

[5] Guo H-R, Chiang H-S, Hu H, Lipsitz SR, Monson RR (1997). Arsenic in drinking water and incidence of urinary cancers. *Epidemiology*.

[6] Guo H-R, Lipsitz SR, Hu H, Monson RR (1998). Using ecological data to estimate a regression model for individual data: the association between arsenic in drinking water and incidence of skin cancer. *Environmental Research*.

[7] Hopenhayn-Rich C, Biggs ML, Smith AH (1998). Lung and kidney cancer mortality associated with arsenic in drinking water in Cordoba, Argentina. *International Journal of Epidemiology*.

[8] IARC (International Agency for Research on Cancer) (1980). *Some Metals and Metallic Compounds (IARC Monographs on the Evaluation of Carcinogenic Risk To HumansInternational Agency For Research on Cancer*.

[9] IARC (International Agency for Research on Cancer) (2004). *Some Metals and Metallic Compounds (IARC Monographs on the Evaluation of Carcinogenic Risk To Humans*.

[10] Huang CF, Chen YW, Yang CY, Tsai KS, Yang RS, Liu SH (2011). Arsenic and diabetes: current perspectives. *Kaohsiung Journal of Medical Sciences*.

[11] Kile ML, Christiani DC (2008). Environmental arsenic exposure and diabetes. *Journal of the American Medical Association*.

[12] Lai M-S, Hsueh Y-M, Chen C-J (1994). Ingested inorganic arsenic and prevalence of diabetes mellitus. *American Journal of Epidemiology*.

[13] Rahman M, Tondel M, Ahmad SA, Axelson O (1998). Diabetes mellitus associated with arsenic exposure in Bangladesh. *American Journal of Epidemiology*.

[14] Coronado-González JA, Del Razo LM, García-Vargas G, Sanmiguel-Salazar F, Escobedo-de la Peña J (2007). Inorganic arsenic exposure and type 2 diabetes mellitus in Mexico. *Environmental Research*.

[15] Lewis DR, Southwick JW, Ouellet-Hellstrom R, Rench J, Calderon RL (1999). Drinking water arsenic in Utah: a cohort mortality study. *Environmental Health Perspectives*.

[16] Ruiz-Navarro ML, Navarro-Alarcón M, Lopez Ga-De La Serrana H, Pérez-Valero V, López-Martinez MC (1998). Urine arsenic concentrations in healthy adults as indicators of environmental contamination: relation with some pathologies. *Science of the Total Environment*.

[17] Ward NI, Pim B (1984). Trace element concentrations in blood plasma from diabetic patients and normal individuals. *Biological Trace Element Research*.

[18] Luu TTG, Sthiannopkao S, Kim K-W (2009). Arsenic and other trace elements contamination in groundwater and a risk assessment study for the residents in the Kandal Province of Cambodia. *Environment International*.

[19] American Diabetes Association (2012). Diagnosis and classification of diabetes mellitus. *Diabet Care*.

[20] Marchiset-Ferlay N, Savanovitch C, Sauvant-Rochat M-P (2012). What is the best biomarker to assess arsenic exposure via drinking water?. *Environment International*.

[21] Del Razo LM, García-Vargas GG, Valenzuela OL (2011). Exposure to arsenic in drinking water is associated with increased prevalence of diabetes: a cross-sectional study in the Zimapán and Lagunera regions in Mexico. *Environmental Health: A Global Access Science Source*.

[22] Kim NH, Mason CC, Nelson RG, Afton SE, Essader AS, Medlin JE (2013). Arsenic exposure and incidence of type 2 diabetes in Southwestern American Indians. *American Journal of Epidemiology*.

[23] Kim Y, Lee B-K (2011). Association between urinary arsenic and diabetes mellitus in the Korean general population according to KNHANES 2008. *Science of the Total Environment*.

[24] Navas-Acien A, Silbergeld EK, Pastor-Barriuso R, Guallar E (2008). Arsenic exposure and prevalence of type 2 diabetes in US adults. *Journal of the American Medical Association*.

[25] Navas-Acien A, Silbergeld EK, Pastor-Barriuso R, Guallar E (2009). Rejoinder: Arsenic exposure and prevalence of type 2 diabetes updated findings from the national health nutrition and examination survey, 2003–2006. *Epidemiology*.

[26] Ettinger AS, Zota AR, Amarasiriwardene CJ (2009). Maternal arsenic exposure and impaired glucose tolerance during pregnancy. *Environmental Health Perspectives*.

[27] Islam MR, Khan I, Hassan SMN, McEvoy M, D'Este C, Attia J (2012). Association between type 2 diabetes and chronic arsenic exposure in drinking water: a cross sectional study in Bangladesh. *Environmental Health*.

[28] Jovanovic D, Rasic-Milutinovic Z, Paunovic K, Jakovljevic B, Plavsic S, Milosevic J (2013). Low levels of arsenic in drinking water and type 2 diabetes in Middle Banat region, serbia. *International Journal of Hygiene and Environmental Health*.

[29] Khan I, Hassan S, McEvoy M, D'Este C, Attia J, Peel R (2011). Association between type 2 diabetes and chronic arsenic exposure in Bangladesh. *Epidemiology*.

[30] Makris KC, Christophi CA, Paisi M, Ettinger AS (2012). A preliminary assessment of low level arsenicexposure and diabetes mellitus in Cyprus. *BMC Public Health*.

[31] Navas-Acien A, Silbergeld EK, Pastor-Barriuso R, Guallar E (2009). Rejoinder: Arsenic exposure and prevalence of type 2 diabetes updated findings from the national health nutrition and examination survey, 2003–2006. *Epidemiology*.

[32] Rahman M, Wingren G, Axelson O (1996). Diabetes mellitus among swedish art glass workers—an effect of arsenic exposure?. *Scandinavian Journal of Work, Environment & Health*.

[33] Milton AH, Hasan Z, Rahman A, Rahman M (2003). Non-cancer effects of chronic arsenicosis in Bangladesh: preliminary results. *Journal of Environmental Science and Health A Toxic/Hazardous Substances and Environmental Engineering*.

[34] Paul S, Das N, Bhattacharjee P, Banerjee M, Das JK, Sarma N (2013). Arsenic-induced toxicity and carcinogenicity: a two-wave cross-sectional study in arsenicosis individuals in West Bengal, India. *Journal of Exposure Science and Environmental Epidemiology*.

[35] Rana T, Bera AK, Bhattacharya D, Das S, Pan D, Das SK (2012). Chronic arsenicosis in goats with special reference to its exposure, excretion and deposition in an arsenic contaminated zone. *Environ Toxicol Pharmacol*.

